# Second Re-irradiation of Brain Metastases: A Review of Studies Involving Stereotactic Radiosurgery

**DOI:** 10.7759/cureus.3712

**Published:** 2018-12-11

**Authors:** Carsten Nieder, Rosalba Yobuta, Bård Mannsåker

**Affiliations:** 1 Oncology, Nordland Hospital Trust, Bodø, NOR

**Keywords:** brain metastases, re-irradiation, stereotactic radiotherapy, radiation therapy, radionecrosis, local control, recurrent brain metastases

## Abstract

Due to advances in the systemic and local treatment, e.g., targeted therapy, immune checkpoint inhibitors, and stereotactic radiotherapy, an increasing proportion of patients with brain metastases now survive for several years. However, long-term survival is not synonymous to permanent local control in the brain. Both local and distant brain relapse sometimes necessitate additional radiotherapy to prevent death from neurologic causes. Prescribing more than two courses of radiotherapy to the same target volume or, in this case, brain metastasis, is a controversial approach. The present review summarizes the results of clinical studies, that included patients treated with whole-brain radiotherapy (WBRT) and two courses of stereotactic radiotherapy to the same, locally recurrent metastasis, and with two courses of WBRT and an additional stereotactic radiotherapy.

## Introduction and background

Fundamental progress in systemic and local treatment, e.g., targeted therapy, immune checkpoint inhibitors, and stereotactic radiotherapy [1-7], has resulted in improvement of long-term survival after the diagnosis of brain metastases, a condition historically associated with a median survival of only a few months [8-9]. Mainly, these advances have been realized through spatial cooperation (extracranially active drugs, local brain radiotherapy); however, an increasing number of drugs is also active in the brain. Survival estimates, though not perfectly accurate for each individual patient, are provided by several prognostic tools, which may assist in clinical decision making [10]. Importantly, long-term survival is not synonymous to permanent local control of the brain metastases. Both local and distant brain relapse can occur after variable time intervals, resulting in the demand for additional radiotherapy, if death from neurologic causes is to be prevented. Prescribing more than two courses of radiotherapy to the same target volume or, in this case, a brain lesion is a controversial approach [11-13]. Opponents have questioned the radiosensitivity of recurrent tumors and pointed toward a lack of consensus regarding the dose constraints for organs at risk (safety concerns). Nevertheless, several clinicians who offered such treatment have published their experience. The present review summarizes the results of clinical studies, which included patients treated with whole-brain radiotherapy (WBRT) and two courses of stereotactic radiotherapy (SRT) to the same, locally recurrent metastasis, and with two courses of WBRT and additional SRT.

## Review

The study is based on a systematic literature search using PubMed and Embase. It is limited to adult patients having received treatment for parenchymal brain metastases. The keywords used were “brain metastases”, “metastatic brain tumor”, "secondary brain tumor" and “cerebral metastases” in combination with "reirradiation", "re-irradiation", “repeat radiotherapy” and “salvage”. The keywords were applied in the same manner, and for all variations in both databases, one combination at a time, e.g. [brain metastases AND reirradiation] or [cerebral metastases AND re-irradiation]. The time interval was 2005-2018, and the final search was performed on August 31, 2018. It also included the reference lists of all articles and the appropriate chapters in textbooks on brain metastases, neuro-oncology, and radiation oncology. Only studies with a minimum number of five patients were included in the review.

We identified and reviewed six retrospective clinical studies of sequential WBRT and two courses of SRT [14-19]. Balermpas et al. reported on 31 patients with 32 recurrent brain metastases (31% each with breast and non-small cell lung cancer [NSCLC]) [14]. Even if the median number of lesions treated in both SRT series was one, up to 10 lesions were irradiated in the initial course and up to three in the second one. The median planning target volume (PTV) size was 2.0 and 2.5 cc, respectively. The median time interval was 12 months (minimum three months). Five patients also received WBRT, i.e., three courses in total, and formed the group of interest, as displayed in Table 1. However, the detailed results were not reported for these individual patients. For all patients, the median follow-up was 11.9 months. One-year survival and local control was 62% and 80%, respectively. Twenty-five patients experienced no toxicity, while five had radiological signs of necrosis. 

**Table 1:** Studies included in the review SRS: stereotactic radiosurgery, WBRT: whole brain radiotherapy, SFRT: stereotactic fractionated radiotherapy, GTV: gross tumor volume *selection criteria not specified

Authors	Number of patients	Sequence	Treatment details	Local failure outcome	Toxicity
Balermpas et al. [14]	4	WBRT+SRS+SRS	SFRT if GTV >3 cm	Not reported	1 Radionecrosis
	1	SRS+SRS+WBRT			
Kim et al. [15]	6	SRS+SRS+WBRT	Not reported	Not reported	Not reported
Koffer et al. [16]	8	WBRT+SRS+SRS	No SFRT	Previous WBRT increased local failure risk (trend)	3 Radionecrosis
McKay et al. [17]	8	WBRT+SRS+SRS	No SFRT	Previous WBRT not sign. associated with local failure	Radionecrosis observed, unknown number
	3	SRS+SRS+WBRT			
Rana et al. [18]	10	WBRT+SRS+SRS	SFRT included*	Previous WBRT not sign. associated with local failure	1 Radionecrosis
	1	SRS+SRS+WBRT			
Moreau et al. [19]	22	WBRT+SRS+SRS	No SFRT	Previous WBRT decreased local failure risk (*p *= 0.003)	4 Radionecrosis
	2	SRS+SRS+WBRT			
Son et al. [20]	5	WBRT+SRS+WBRT	Not reported	Not reported	1 Radionecrosis
	6	WBRT+WBRT+SRS			
Ozgen et al. [21]	10	WBRT+SRS+WBRT	No SFRT	Not reported	No radionecrosis

Kim et al. [15] included 114 patients with 176 locally recurrent metastases (59% NSCLC). Eighty percent had a single recurring metastasis. For the few patients who received WBRT as their third course, no separate outcome data were provided. By contrast, Koffer et al. [16] reported more details about their eight patients who received additional WBRT. Overall, their study included 22 patients with 24 radiosurgery (SRS)-managed lesions (41% NSCLC). The median target volume size was 2.25 and 3.3 cc, respectively, while the median dose was 18 and 15.5 Gy, respectively. The median time interval was 13.4 months (median follow-up was 8.8 months, identical to the median survival). One-year survival and local control was 38% and 61%, respectively. Out of eight patients, three each developed local failure or radionecrosis (75% combined), meaning that only 25% achieved an optimal outcome. Stratified by prior WBRT, local failure rates were 37.5% and 12.5%, respectively (*p* = 0.15). The corresponding figures for radionecrosis were 37.5 and 6.3% (*p *= 0.05), respectively.

The fourth eligible study included 32 patients with 46 lesions treated with repeated SRS (50% NSCLC) [17]. The median time interval was 19 months (minimum two months). The median tumor volume was 1.3 and 1 cc, respectively (median dose 20 + 20 Gy). One-year survival and local control was 70% and 79%, respectively (not available for the subgroup of interest). Prior WBRT was not significantly associated with local failure (neither with radionecrosis). However, details and percentages were not reported. Based on all 46 lesions, the rate of radiation necrosis was 30%.

The fifth eligible study included 11 patients of interest (a total number of 28 patients with 32 metastases, mainly melanoma [39%]) [18]. The median gross tumor volume (GTV) size was 0.5 and 1.4 cc, respectively and the median dose was 24 and 26.5 Gy, respectively. The median time interval was 10 months. In case of previous WBRT, the median time to first SRT was 11 months (minimum three months). One-year survival and local control was 91% and 88%, respectively (not available for the patients of interest). Prior WBRT was not significantly associated with local failure (neither with radionecrosis). However, details and percentages were not reported. Based on all 32 lesions, six (18.8%) developed radionecrosis. 

Moreau et al. [19] have published the largest dataset of interest (*n *= 24), extracted from their analysis of 30 patients with 36 lesions (50% lung cancer, not stratified by histology). None of the patients had a neurological deficit at the time of second SRS. Minimum Karnofsky performance status (KPS) was 70. The maximum diameter of local recurrence was 3 cm and it was located >5 mm away from the brainstem, optic nerves, chiasm, and outside the motor area. The median PTV size was 1.5 and 4.8 cc, and the median dose was 18 Gy at the isocenter (all 30 patients). Median follow-up from final SRS was 14 months. WBRT was standardized, always 10 fractions of 3 Gy. For all 30 patients, one-year local control was 68%. Previous WBRT was associated with a better local control (hazard ratio: 0.25, 0.1 to 0.64, *p *= 0.003). One-year survival was 66%. Four out of the 22 patients (18%) with initial WBRT developed radionecrosis, five hemorrhages, and five radiologic signs of edema. These patients did not develop neurologic deficits (Radiation Therapy Oncology Group (RTOG) grade one or two adverse effects).

Two additional studies involved patients managed with repeated WBRT rather than repeated SRS. Son et al. reported on 17 patients treated with repeated WBRT, 11 of whom also received SRS [20]. The median WBRT dose was 35 and 21.6 Gy, respectively. The median time interval between the WBRT series was 18 months (minimum 3.6 months). Median survival after second WBRT was 5.0 months. Unfortunately, detailed outcomes for the patients of interest are lacking. Ozgen et al. reported on 28 patients treated with repeated WBRT, 10 of whom also received SRS [21]. The median WBRT dose was 30 and 25 Gy, respectively. The median SRS dose was 14 Gy. The median time interval from WBRT to SRS was 11 months (minimum four months), and the median interval from SRS to second WBRT was eight months (minimum four months). Median survival after second WBRT was three months (not significantly different if SRS had been given). Symptomatic response after second WBRT was relatively uncommon if SRS also had been administered (30% vs. 64% without previous SRS, *p *= 0.36).

**Figure 1:**
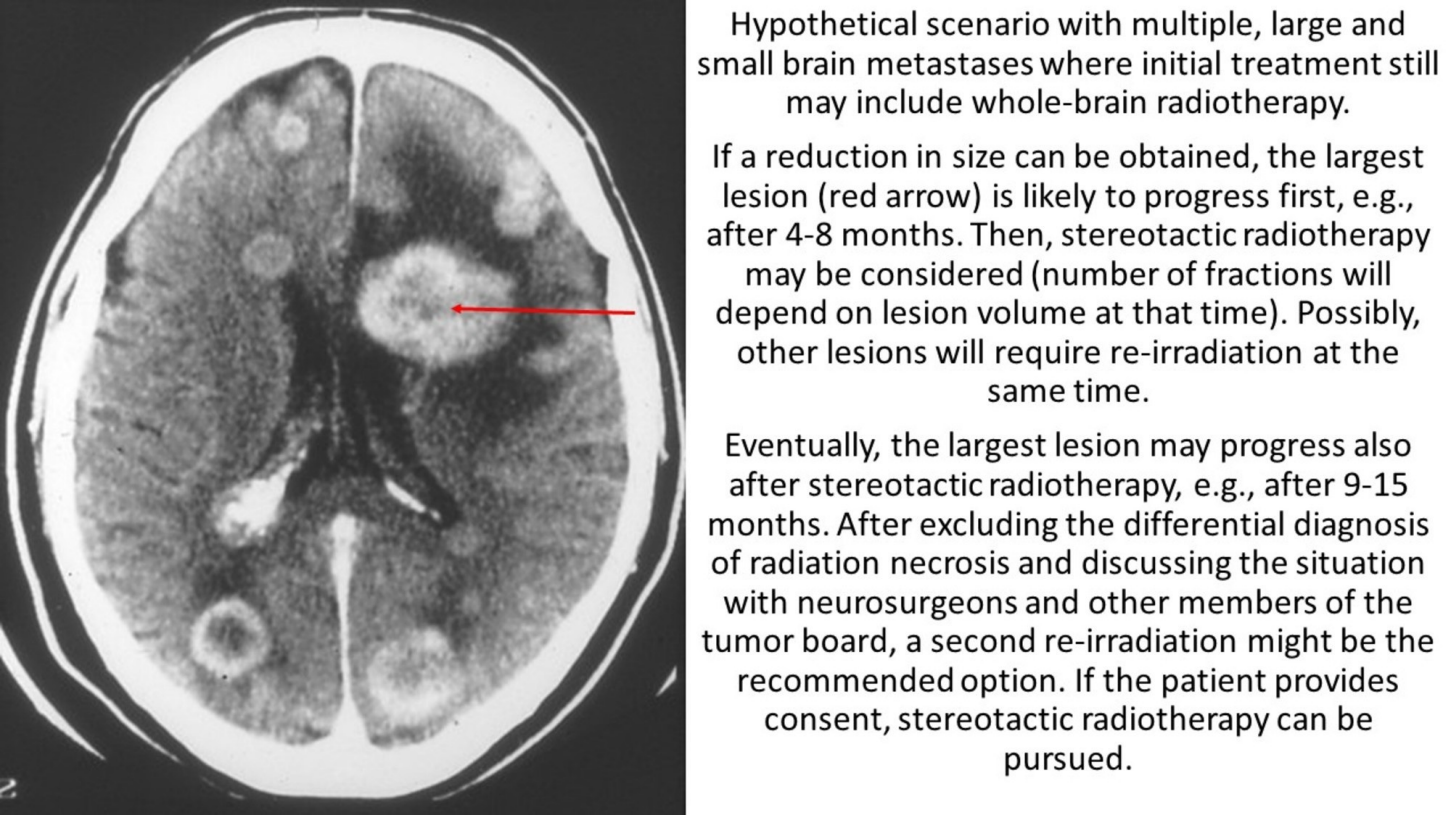
Scenario with initial whole-brain radiotherapy

The present review did not identify any large and/or prospective clinical studies. Summarized, the patient numbers ranged from five to 24. For the first scenario (two series of SRT and one WBRT), six studies were eligible [14-19]. The typical sequence of radiotherapy (RT) was WBRT first, followed by SRT (overall 52 patients), while 13 patients finished their RT sequence with WBRT. Figure 1 shows a hypothetical patient case. 

Most studies failed to provide useful data for the patients treated with three courses. Only one reported all the time intervals between the three series (10 and 11 months) [18]. The others reported only one interval (median: 10, 12, 13, and 19 months). Occasionally, patients were re-irradiated after two or three months. Only one study reported the WBRT dose/fractionation regimen [19]. Thus, the dose recommendations or constraints cannot be provided. Likewise, an individual patient data meta-analysis cannot be performed. Median follow-up was not reported in all studies (if available, maximum 14 months). The one-year survival rates were reported for all patients rather than those who were treated with three courses. It ranged from 38% to 91%, with the median being 62%. Except for one study with eight patients [16], local control was only reported for all study patients and not those of interest. The one-year local control rates ranged from 68% to 88% (median 79%). However, it was lower in the one study with separate data (62.5%). These conflicting data indicated that initial WBRT could either decrease or increase the risk of local failure, while two studies found no significant association. However, it is important to emphasize that the statistical power of these studies was low, meaning that clinically important differences were unlikely to be detected. In the study with the best quality of data reporting (and at the same time largest size) [19], the rate of radionecrosis was 18% after WBRT and two courses of SRS. The other studies reported 10% to 37.5%. If pooled, the overall rate was 20% (nine of 46 patients). This figure is difficult to put in context due to the lack of dosimetric and volumetric information. Moreover, it remains unknown if the risk was higher in patients re-irradiated after shorter time intervals or given potentially sensitizing systemic treatment. Nevertheless, one would not expect radionecrosis rates in the order of 20% after just a single SRS treatment, unless large lesions are treated with high-dose single fraction [22-26]. In the setting of brain glioma, SRS re-irradiation after the initial fractionated radiotherapy has resulted in radionecrosis rates of ≥20% [27].

For the second scenario (two courses of WBRT and one SRS), two studies were eligible [20-21]. These small studies reported shorter survival after third RT than the ones included in the first scenario. As only one patient developed radionecrosis, the overall risk appears lower, consistent with the fact that cumulative equivalent dose was lower. The rate of symptomatic response after second WBRT was relatively low (30%). Yet, this result is derived from only 10 patients. Given that many institutions currently delay WBRT and offer two or more courses of SRS or other focal treatments instead, the utilization rates of repeated WBRT are expected to decline. We could not identify data related to neurocognitive function or quality of life in the literature on repeat re-irradiation. Due to the limitations of all studies reviewed here and a lack of information about the causes of death and other relevant endpoints, prospective collection of additional data is necessary before any strong recommendations can be made. In the meantime, individual concepts including repeated SRS and salvage WBRT should be discussed with eligible patients.

## Conclusions

The rates of adverse effects appear not clearly prohibitive when three courses of radiotherapy have to be considered because occasional patients have run out of other options. Eventually, such patients must decide and consent on an individual basis and the present review provides the best available clinical evidence to support decision making, also for clinicians participating in tumor boards where all treatment options should be discussed. The available data is not sufficient to determine how many patients experience local control and/or symptomatic improvement without high-grade treatment-induced adverse events, or in other words, the unequivocal net benefit from three courses of radiotherapy.
